# Accuracy of clinical tests in the diagnosis of anterior cruciate ligament injury: a systematic review

**DOI:** 10.1186/s12998-014-0025-8

**Published:** 2014-08-01

**Authors:** Michael S Swain, Nicholas Henschke, Steven J Kamper, Aron S Downie, Bart W Koes, Chris G Maher

**Affiliations:** 1 The George Institute for Global Health, Sydney Medical School, University of Sydney, Missenden Rd, Sydney, 2050, Australia; 2 Department of Chiropractic, Faculty of Science, Macquarie University, Sydney, 2109, Australia; 3 Institute of Public Health, University of Heidelberg, Heidelberg, 69120, Germany; 4 Department of Epidemiology and Biostatistics, EMGO + Institute, VU University Medical Center, Amsterdam, 1081BT, Netherlands; 5 Department of General Practice, Erasmus MC, University Medical Centre, Rotterdam, 50 3015 GE, Netherlands

**Keywords:** Anterior cruciate ligament, Diagnosis, Medical history taking, Physical examination, Diagnostic test accuracy

## Abstract

**Background:**

Numerous clinical tests are used in the diagnosis of anterior cruciate ligament (ACL) injury but their accuracy is unclear. The purpose of this study is to evaluate the diagnostic accuracy of clinical tests for the diagnosis of ACL injury.

**Methods:**

Study Design: Systematic review. The review protocol was registered through PROSPERO (CRD42012002069).

Electronic databases (PubMed, MEDLINE, EMBASE, CINAHL) were searched up to 19th of June 2013 to identify diagnostic studies comparing the accuracy of clinical tests for ACL injury to an acceptable reference standard (arthroscopy, arthrotomy, or MRI). Risk of bias was appraised using the QUADAS-2 checklist. Index test accuracy was evaluated using a descriptive analysis of paired likelihood ratios and displayed as forest plots.

**Results:**

A total of 285 full-text articles were assessed for eligibility, from which 14 studies were included in this review. Included studies were deemed to be clinically and statistically heterogeneous, so a meta-analysis was not performed. Nine clinical tests from the history (popping sound at time of injury, giving way, effusion, pain, ability to continue activity) and four from physical examination (anterior draw test, Lachman’s test, prone Lachman’s test and pivot shift test) were investigated for diagnostic accuracy. Inspection of positive and negative likelihood ratios indicated that none of the individual tests provide useful diagnostic information in a clinical setting. Most studies were at risk of bias and reported imprecise estimates of diagnostic accuracy.

**Conclusion:**

Despite being widely used and accepted in clinical practice, the results of individual history items or physical tests do not meaningfully change the probability of ACL injury. In contrast combinations of tests have higher diagnostic accuracy; however the most accurate combination of clinical tests remains an area for future research.

**Clinical relevance:**

Clinicians should be aware of the limitations associated with the use of clinical tests for diagnosis of ACL injury.

## Background

The anterior cruciate ligament (ACL) is an important stabilising structure of the knee and its disruption is associated with pain and activity limitation [1]. The annual incidence of ACL injury ranges from 0.01% to 0.05% [2], however it is higher in sporting groups and most frequently affects individuals during late adolescence and early adulthood [3]–[5]. The prevalence of ACL injury in adults presenting to primary care with acute knee pain is estimated to be 4% [6]. Many cases are initially missed [7] in primary care and these undiagnosed ACL injuries are of concern because of the risk of cartilage tear and premature knee osteoarthritis [8].

Clinical diagnosis of ACL injury is based upon history and physical examination findings with suspected cases confirmed by MRI or arthroscopy [9]. Numerous clinical tests and findings have been proposed to aid the diagnosis of ACL injury. A popping sound, swelling and instability following high impact sport trauma along with a positive Lachman’s, anterior draw or pivot shift test is the most common method of clinical diagnosis [9]. However, there are over 25 specific physical tests and numerous features from the clinical history that have been proposed for detection of ACL injury [10]. At present the diagnostic accuracy of these tests is unclear.

Most existing reviews evaluating the accuracy of tests to diagnose ACL injury [6],[11]–[14] are now over a decade old and contain methodological limitations such as inclusion of inappropriate studies and pooling of estimates from heterogeneous studies. Since these reviews were published there has been much progress in the diagnostic field with regard to study appraisal and synthesis [15]. There is now a greater appreciation of how design features may lead to biased estimates of diagnostic test accuracy and when meta-analysis is justified. In addition it is likely that more recent primary research studies have been conducted in the area of ACL diagnosis.

The objective of this systematic review was to report the diagnostic accuracy of clinical tests for the diagnosis of ACL injury and describe the quality of research evaluating these tests.

## Methods

A systematic review protocol [16] was registered at the International Prospective Register of Systematic Reviews -PROSPERO 2012:CRD42012002069.

### Identification of selected studies

Electronic databases (PubMed, MEDLINE, EMBASE, and CINAHL) were searched for eligible diagnostic studies from the earliest year possible up to 19th of June 2013. The search strategy was developed for PubMed and modified for use in other databases (Additional file 1: Table S1). The reference lists of all included publications and relevant systematic reviews were checked and forward citation searches performed.

### Eligibility criteria

Diagnostic studies were eligible if they compared the accuracy of history taking or physical examination to an acceptable reference standard (arthroscopy, arthrotomy, or MRI) in the identification of ACL injury. Both prospective and retrospective studies were eligible for inclusion. We did not include case control studies as they substantially overestimate diagnostic accuracy compared with studies that use a clinical population [17].

The focus of this review was on studies that evaluated patients presenting to a care provider for diagnosis of knee pain or dysfunction, where the diagnostic accuracy of individual, or combinations of, history features or physical assessment procedures was evaluated. Studies in which a substantial proportion of recruited patients had already been diagnosed with ACL injury were excluded to minimise verification bias [17].

Included studies had to report sufficient data on diagnostic tests to enable construction of a 2 × 2 table so estimates of diagnostic accuracy (such as sensitivity and specificity) could be calculated. Studies that evaluated the accuracy of an unspecified combination of history and physical examination, such as clinical diagnosis or global clinician judgment were excluded as they do not allow for replication, validation and generalization of the study results [18].

If studies had been reported in abstracts or conference proceedings, the related full publications were retrieved if available, but only full articles published in peer-reviewed journals were included. Studies published in all languages were considered eligible and translations were sought where necessary.

### Study selection

Two authors (MS and NH) independently screened all titles and abstracts identified in the searches with respect to the inclusion and exclusion criteria. Full text copies of potentially relevant articles were retrieved and final inclusion or exclusion was determined. Disagreements regarding inclusion were resolved by consensus, including a third review author (SK) where necessary.

### Data extraction

Three review authors (MS, NH, SK) independently extracted information from the included studies. Data were extracted into a specifically designed spreadsheet and included details on the study design, setting, enrolment procedures, number of participants, patient demographics, and time since initial ACL injury. Details of the type of index test and the type of reference standard were also extracted and the proportion of participants with ACL injuries was calculated for each included study. Diagnostic two-by-two tables (true positive, false positive, true negative and false negative) were either extracted from the publications or reconstructed using information from other reported parameters (sensitivity, specificity, or predictive values). Uninterpretable index test outcomes, such as an equivocal finding were dealt with as a negative index test finding. The authors of one study [19] were contacted and provided additional information.

### Quality assessment

The quality of each included study was assessed by two review authors (MS, NH) using the QUality Assessment of Diagnostic Accuracy Studies (QUADAS-2) checklist [20]. The QUADAS-2 checklist consists of four domains relating to patient selection, index test, reference standard, and flow and timing. Each domain is assessed in terms of risk of bias, and the first 3 domains are also assessed in terms of applicability. The review authors classified each item as “yes” (adequately addressed), “no” (inadequately addressed), or “unclear” (inadequate detail presented to allow a judgment to be made). Disagreements were resolved by consensus and consulting with a third (SK) review author where necessary.

### Synthesis of results

The two-by-two tables were used to calculate index test summary statistics: sensitivity, specificity, likelihood ratios along with their 95% confidence intervals using MetaDiSc 1.4. Index test accuracy was presented as forest plots of likelihood ratios, as likelihood ratios provide the best way for clinicians to use diagnostic data to establish clinical diagnoses in patient care [21]. Categorisation of likelihood ratios was adopted from Jaeschke et al. [18] where positive likelihood ratios (+LR) <5 and a negative likelihood ratios (−LR) >0.2 were considered small, +LR 5–10 and –LR 0.1-0.2 were moderate, and + LR>10 and –LR <0.1 were considered large, with respect to changing the pre to post-test probability.

Both clinical and statistical heterogeneity as well as methodological quality were evaluated to determine the appropriateness of meta-analysis. Assessment of clinical heterogeneity involved comparison of the study populations, settings, performance of index tests and reference standards among included studies. Assessment of statistical heterogeneity involved visual inspection of forest plots and performance of the chi-square (χ^2^) test and calculation of the inconsistency index (I^2^) which quantifies the proportion of variation across the included studies that is due to heterogeneity rather than chance [22].

## Results

### Study selection

**Figure 1:**
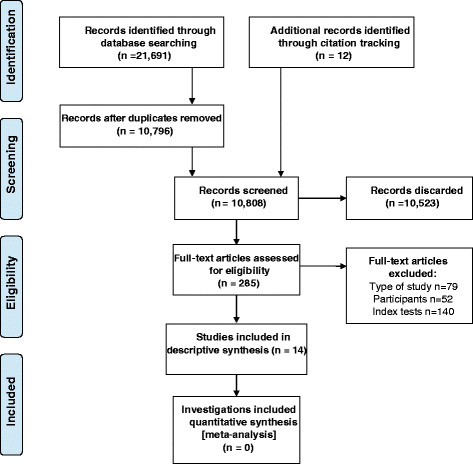
PRISMA flow diagram of studies through the review.

The initial database searches retrieved 21,691 citations of which 10,796 citations remained after duplicates were removed (Figure 1). Screening of the titles and abstracts identified 285 potentially relevant articles that were retrieved in full text format. Forwards and backwards citation tracking identified 12 potentially relevant articles which were also retrieved. Fourteen articles were finally included, of which 11 were published in English [19],[23]–[32] and three in German [33]–[35]. Additional file 2: Table S2 lists the reasons for excluding 28 articles that were included in one or more of the previous five systematic reviews.

### Description of included studies

Of the 14 included articles, 10 had a prospective study design [19],[23],[24],[28]–[30],[32]–[35], two used a retrospective design [26],[27] and for two studies [25],[31] the design was unclear (Table 1).

**Table 1:** Characteristics of included studies

**First author, year**	**Design**	**Setting**	**Participants**	**Partial** ***and*** **complete ACL tear prevalence % (n)**	**Complete ACL tear prevalence % (n)**	**Reference standard(s)**
Beldame, 2011 [23]	Prospective	University hospital, France.	*112 patient/knees with an indication for knee arthroscopy.	37.5% (42)	28.5% (32)	Arthroscopy
Boeree, 1991 [24]	Prospective	Orthopaedic clinic, UK.	203 patient/knees referred from GPs or the A&E.	29.1% (51)	nr	MRI
Decker, 1988 [33]	Prospective	Hospital, Germany.	†108 patient/knees suspected to have knee ligament injury.	61.1% (66)	nr	Arthroscopy/Surgery
Harilainen, 1987 [25]	Unclear	Emergency department, Finland.	†350 patient/knees with acute knee injury.	41.7% (146)	nr	Arthroscopy/Arthrotomy
Katz, 1986 [26]	Retrospective	Community hospital, USA.	85 participant/knees with knee injuries presenting for arthroscopy.	25.9% (22)	nr	Arthroscopy
Lee, 1988 [27]	Retrospective	Orthopaedic department of a hospital, USA.	79 magnetic resonance studies of the knee were reviewed.	29.1% (22)	nr	MRI
Lucie, 1984 [28]	Prospective	Orthopaedic clinic, USA.	50 patient/knees with acute traumatic knee haemarthrosis.	76.0% (38)	nr	Arthroscopy/Arthrotomy
Mulligan, 2011 [29]	Prospective	Orthopaedic surgery and sports medicine service, USA.	*†52 patient/knees with a complaint of knee pain referred from emergency department.	44.2% (23)	nr	Arthroscopy
Noyes, 1980 [30]	Prospective	Orthopaedic/Sports medicine knee clinic, USA.	*85 injured knees (83 patients) that had traumatic haemarthrosis.	71.8% (61)	43.5% (37)	Arthroscopy
Richter, 1996 [34]	Prospective	Hospital, Germany.	74 patient/knees with effusion of the knee following trauma.	78.4% (58)	64.9% (48)	Arthroscopy
Schwartz, 1997 [35]	Prospective	Hospital, Germany.	58 patient/knees with acute knee injury.	81.0% (47)	65.5% (38)	Arthroscopy
Tonino, 1986 [31]	Unclear	Hospital, Netherlands.	*66 patient/knees with acute symptoms of a ligamentous lesion of the knee after trauma.	45.5% (30)	nr	Arthroscopy
Wagemakers, 2010 [19]	Prospective	GP clinics, Netherlands.	*134 patient/knees with new knee symptoms.	20.9% (28)	12.7% (17)	MRI
Wong, 1999 [32]	Prospective	Orthopaedic department of a hospital, Hong Kong.	91 patient/knees with an acute knee haemarthrosis.	nr	56.0% (51)	Arthroscopy

nr: not reported.

*Not all participants evaluated by index test(s).

†Not all participants evaluated by reference standard.

Only one study [19] evaluated the diagnostic accuracy of clinical tests in primary care. The other 13 studies evaluated the accuracy of clinical tests in secondary contact settings, defined here as either a referral to an orthopaedic department or presentation to an accident and emergency department. In three studies the reference standard was MRI [19],[24],[27], eight studies applied arthroscopy [23],[26],[29]–[32],[34],[35] and three studies applied either arthroscopy or arthrotomy [25],[28],[33]. Only five studies [25]–[27],[29],[30] reported in detail the method of index test application with slight variations between them in the way the index tests was performed.

Nine studies [19],[23],[26],[28],[30]–[32],[34],[35] assessed diagnostic accuracy for partial or complete ACL injuries, however only four of these [19],[23],[30],[32] provided sufficient information to determine if the index test result pertained to a partial or complete disruption of the ACL. Injury severity (partial or complete ACL disruption) was unclear and treated as partial *and* complete injuries in the remaining studies. Nine studies [19],[24]–[26],[28],[30],[31],[33],[35] described ACL injuries with concomitant injury to other knee structures, while comorbid knee injuries were unclear or not reported in the remaining five studies [23],[27],[29],[32],[34].

There was variability between participants in the included studies with respect to sample size (50–350), average age (25–40 years), proportion of males (52%-100%) and time since ACL injury (one day to longer than one year). The prevalence of verified partial *and* complete ACL injury ranged from 21%-81%.

### Quality assessment

The QUADAS-2 ratings of risk of bias and study applicability are shown in Table 2. Only one study [19] adequately addressed all risk of bias domains. For the 14 studies, risk of bias was high or unclear with regard to patient selection for 10 studies, for the index text four studies, for the reference standard nine studies and for flow and timing eight studies.

**Table 2:** Risk of bias and applicability concerns summary based on the QUADAS-2 checklist

	**Risk of bias**				**Applicability concerns concerns**		
**First author, year**	**Patient selection**	**Index test**	**Reference standard**	**Flow and timing**	**Patient selection**	**Index test**	**Reference standard**
Beldame, 2011 [23]	?	+	+	-	+	+	+
Boeree, 1991 [24]	-	?	+	+	+	+	+
Decker, 1988 [33]	?	+	?	-	+	+	+
Harilainen, 1987 [25]	+	?	?	-	+	+	+
Katz, 1986 [26]	-	+	?	+	+	+	+
Lee, 1988 [27]	-	+	?	-	+	+	+
Lucie, 1984 [28]	-	+	?	-	+	+	+
Mulligan, 2011 [29]	+	+	?	?	+	+	+
Noyes, 1980 [30]	-	?	+	-	?	+	+
Richter, 1996 [34]	-	+	?	+	+	+	+
Schwartz, 1997 [35]	-	+	+	+	+	+	+
Tonino, 1986 [31]	-	+	?	?	+	+	+
Wagemakers, 2010 [19]	+	+	+	+	+	+	+
Wong, 1999 [32]	+	?	?	+	+	+	+

Judgements on risk of bias and applicability concerns: − = high risk; ? = unclear risk; + = low risk.

Only one study [19] clearly stated that the reference standard was assessed without knowledge of the results of the index test, while in 12 studies this was unclear [23]–[26],[28]–[35]. In one study the reference standard was not applied independently of clinical tests [27]. Six studies [24],[27],[28],[32],[34],[35] included all enrolled participants in the analysis. Across the remaining eight studies [19],[23],[25],[26],[29]–[31],[33] the number of participants left out of the analyses ranged from 1%-71% of those originally included.

### Diagnostic accuracy of clinical tests

**Figure 2:**
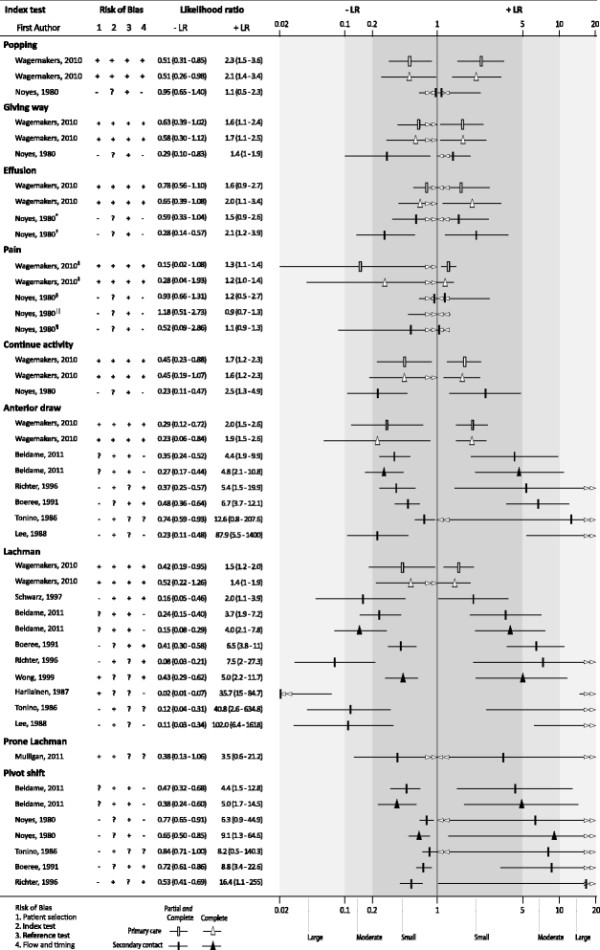
**Diagnostic accuracy of clinical examination for ACL injury.** Legend: Risk of bias judgements: (−) = high risk; (?) = unclear risk; (+) = low risk. LR thresholds: +LR <5 and -LR >0.2 = small; +LR 5–10 and; −LR 0.1–0.2 = moderate and +LR >10 and –LR <1 = large. Studies that reported estimates for complete ACL injury as well as partial *and* complete ACL injury estimates have been plotted together to provide a comparison of test performance. Different symbols are used for the estimates for complete versus partial and complete ACL injury and for primary care versus secondary contact settings. *joint effusion 2 hours; †joint effusion 12 hours; ‡immediate pain at trauma; §pain none to slight; ||pain moderate to severe; ¶guarded or painful ROM 24 hours after injury. Guide for interpretation: Greater distance between the –LR and +LR symbols for the test indicates better diagnostic performance.

A total of nine clinical index tests were identified by this review. Five tests were items from the clinical history (popping sound at time of injury, giving way, effusion, pain, ability to continue activity) and four index tests were applied as part of physical assessment (the anterior draw test, Lachman’s test, prone Lachman’s test, the pivot shift). Three of the tests were also performed under anaesthesia (anterior draw test, Lachman’s test, pivot shift test). Diagnostic accuracy statistics for all index tests are presented as supplemental material (Additional file 3: Table S3). The anterior draw, Lachman and pivot shift tests were each evaluated in subgroups where the tests were applied in secondary contact settings to identified partial *and* complete ACL injury. The chi-square test ranged from χ^2^ = 50.66, 6df, P < 0.001 to χ^2^ = 6.55, 4df, P = 0.16 and the inconsistency indexes were typically high (>75%) ranging from 99.2% to 38.9%. The three physical tests plotted on the ROC plane as well the subgroups sensitivity and specificity forest plots are presented as supplementary information (Additional file 4: Figure S1, Additional file 5: Figure S2). The variability in patient spectrum and performance of index tests among the included studies resulted in important clinical and statistical heterogeneity. In addition, only a small number of studies evaluate specific clinical tests, with all but one study at high risk of bias, so a decision was made not to perform a meta-analysis. The diagnostic accuracy of individual clinical tests for ACL injury along with thresholds for defining clinical usefulness (i.e. small, moderate and large change in post-test probability) are illustrated in Figure 2. The number of studies that evaluated each individual test ranged from two studies for clinical history items to nine studies for Lachman’s test.

Only two studies [19],[30], from different settings (primary and secondary care), investigated test accuracy for clinical history items. Clinical history items had low value in correctly diagnosing ACL injury (+LR range 0.93-2.54, −LR range 0.15-1.18) (Figure 2).

Six studies [19],[23],[24],[27],[31],[34] reported the accuracy of the anterior draw test in diagnosing ACL injury. Small, moderate and large +LR (range 1.94-87.88) were observed for the anterior draw test across studies. The large +LR estimates all had wide confidence intervals and were reported in studies with high risk of bias. All –LRs (range 0.23-0.74) for the anterior draw test were within the small threshold.

Nine studies [19],[23]–[25],[27],[31],[32],[34],[35] investigated the accuracy of Lachman’s test in diagnosing ACL injury. Small, moderate and large LRs (+LR range 1.39-40.81, −LR range 0.02-0.52) were reported for Lachman’s test across the studies. Studies that report moderate or large LRs tended to be at risk of bias and had wide confidence intervals. One study [29] investigated the prone Lachman’s test and reported small and imprecise LRs (+LR 3.50, −LR 0.38).

Five studies [23],[24],[30],[31],[34], all at risk of bias, evaluated the accuracy of the pivot shift test. Small, moderate and large +LRs (range 4.37-16.42) and small –LRs (range 0.38-0.84) were reported for the pivot shift test in all studies. Accuracy estimates with moderate and large +LRs tended to lack precision.

Five studies at high risk of bias [26],[28],[30],[31],[33] investigated physical tests when examination was performed under anaesthesia (EUA) (Additional file 6: Figure S3). The anterior draw test, Lachman’s test and pivot shift test appear to provide improved diagnostic accuracy when examination is performed under anaesthesia. While LRs are moderate-large the confidence intervals around the +LR estimates are wide.

**Figure 3:**
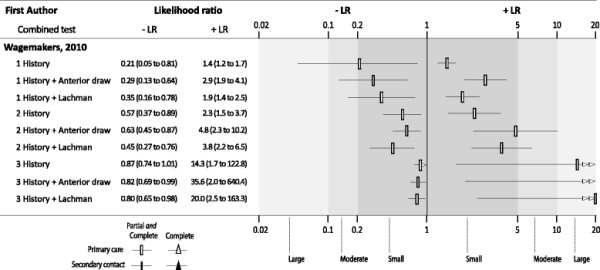
**Diagnostic accuracy of composite index tests for partial** ***and*** **complete ACL injury in primary care.** Legend: Thresholds: +LR <5 and -LR >0.2 = small; +LR 5–10 and; −LR 0.1–0.2 = moderate and +LR >10 and -LR <1 = large. Guide for interpretation: Greater distance between the –LR and +LR symbols for the test indicates better diagnostic performance.

Only one study, from the primary care setting with low risk of bias, provided data on the effect of combining clinical tests [19]. Specifically, this included two or three positive history tests (from a list of popping sensation, giving way, effusion, immediate pain at trauma and continuation of activity impossible) as well as a positive anterior draw or Lachman’s test (Figure 3). The addition of a positive anterior draw test to the combinations of two positive history tests increase the +LR (4.81) close to moderate diagnostic threshold. The addition of a third history test produced a large but imprecise +LR (35.64) but reduced the –LR (0.82).

## Discussion

This systematic review included 14 studies that evaluated the diagnostic accuracy of clinical tests for ACL injury. Just one study, which was the only study performed in primary care, had a low risk of bias and showed that results of individual tests produce only small changes in the probability of ACL injury. The same study investigated the diagnostic accuracy of combining history items with physical tests and reported improved accuracy when doing so. The other studies, performed in secondary contact settings, had moderate to high risk of bias and reported quite diverse and imprecise estimates of diagnostic accuracy. Based upon these findings, clinical tests in combination, but not individually, may assist the diagnosis of ACL injury.

The key strengths of the review include a pre-specified and registered review protocol, the use of inclusion criteria to ensure that the study settings reflected clinical practice and the evaluation of study quality using the QUADAS-2 checklist. This review also reported likelihood ratios as they are the preferred approach to report estimates of diagnostic accuracy [21]. The limitations of the study were that sparse data were available on most clinical tests and that we were unable to perform a meta-analysis due to heterogeneity in the estimates of diagnostic accuracy, risk of bias and clinical characteristics. The heterogeneity among studies is well illustrated by the results for Lachman’s test, where reported +LRs ranged from 1.5 to 102, risk of bias varied and ACL injury prevalence in the included studies ranged from 21% to 81%.

The clinical tests reviewed are those most commonly used for the diagnosis of ACL injury in clinical practice. Our findings suggest that a clinician cannot rely on a single clinical test in isolation, particularly one from the clinical history, to identify patients with ACL injury. Due to the fact that diagnostic decisions regarding ACL injury are not made on the basis of a single test, studies should ideally focus on test performance in combination. The best estimates of diagnostic accuracy come from Wagemakers et al. [19] whose data suggest that there may be some potential in combining clinical tests, specifically the anterior draw test with two or three of the following five history findings: popping sensation, giving way, effusion, immediate pain at trauma and inability to continue activity. Notwithstanding, these findings must be interpreted with caution as a major drawback of Wagemakers et al’s study was its low power to sufficiently analyse multiple combined tests. An important direction for future research is identification of the optimal combination of currently available clinical tests to accurately diagnose ACL injury. While the literature regarding the accuracy of currently used tests is of variable quality, those identified in this body of literature (and included in this review) are the logical candidates to investigate using more robust methods. Such studies are well suited to primary care settings (limiting referral bias), but sample sizes will need to be substantially larger than studies to date in order to investigate multiple sequencing of index tests.

In contrast to our findings, previous systematic reviews have concluded that individual clinical tests can be used to accurately diagnose ACL injury [11],[14]. The difference in conclusions is primarily because we only included studies evaluating a clinical sample with diagnostic uncertainty. Other reviews have included case–control studies, a study design which has been shown to inflate estimates of diagnostic accuracy [36]. Our decision to interpret test accuracy via clinically usefully thresholds of likelihood ratios also distinguishes this from previous reviews. A final point of difference concerns our decision not to pool accuracy estimates, which we believe this is the only appropriate course given the risk of bias and heterogeneity evident in the included studies.

Although we applied a critical approach to study selection we still identified several methodological issues that affect internal validity and may result in overestimation of diagnostic test accuracy [17],[37]. The spectrum of patients in the included studies varied because of different methods in patient sampling. Most obviously, the characteristics of the samples varied due to the differences in study inclusion and exclusion criteria. Two recent prospective cohort studies illustrate this: Wagemakers et al. [19] included participants with new knee symptoms and excluded participants who were suspected of knee fracture; whereas Beldame et al. [23] included participants with indication for knee arthroscopy, meaning the sample was subject to referral filter bias [37]. The paucity of diagnostic studies for ACL injury conducted in primary care also suggests caution should be taken when generalising these findings to this setting.

In some instances the index tests were not applied to all participants prior to the application of the reference test, or the reference test was performed without a clinical test. There was under reporting of reasons for patient exclusion and withdrawals. Reporting was deficient in most primary studies which limited our appraisal of study quality. This is perhaps most evident with respect to risk of bias domains associated with blinding of the index tests and reference standards. Where multiple index tests were applied concurrently it is unclear the extent to which knowledge of prior testing (test review bias) overestimated the accuracy of index tests. Similarly, there was concern that the invasive nature of knee arthroscopy or surgery as a reference test may have affected the flow of participants through some studies. In these instances a patient with a negative index test may not have received a reference test creating partial verification bias.

## Conclusion

This systematic review of clinical tests for ACL injury incorporates the most recent knowledge of diagnostic test accuracy methods. The findings highlight the lack of clinical test accuracy data to support the use of history and physical examination to diagnose ACL injury. Most diagnostic studies on this topic contain methodological flaws which can overestimate the diagnostic accuracy of clinical tests. The available high quality evidence suggests that tests are not useful on their own but combinations may prove to be more useful.

## Abbreviations

ACL: Anterior cruciate ligament

QUADAS: QUality assessment of diagnostic accuracy studies

+LR: Positive likelihood ratio

-LR: Negative likelihood ratio

EUA: Examination under anaesthesia

## Competing interests

The authors declare that they have no competing interests.

## Authors’ contributions

Conception and design: MSS, NH, SJK, BWK, CGM. Analysis and interpretation of the data: MSS, NH, SJK, ASD, BWK, CGM. Drafting of the article: MSS, NH, SJK, ASD, CGM. Critical revision of the article for important intellectual content: MSS, NH, SJK, ASD, BWK, CGM. Final approval of the article: MSS, NH, SJK, ASD, BWK, CGM. Statistical expertise: NH, SJK, CGM. Administrative, technical, or logistic support: MSS, NH, SJK, CGM. Collection and assembly of data: MSS, NH, SJK, ASD. All authors read and approved the final manuscript.

## Additional files

## Supplementary Material

Additional file 1: Table S1. PubMed search strategy. Click here for file

Additional file 2: Table S2. Excluded articles from previous systematic reviews. Click here for file

Additional file 3: Table S3. Summary statistics for Index tests in the diagnosis of ACL injury. Click here for file

Additional file 4: Figure S1. Anterior draw, Lachman and pivot shift tests plotted on the ROC plane. Legend: Green: Primary care study setting. Red: Secondary contact study setting. Click here for file

Additional file 5: Figure S2. Subgroup sensitivity and specificity forest plots. Legend: Anterior draw, Lachman and pivot shift test sensitivity and specificity for partial *and* complete ACL injury in secondary contact settings. Click here for file

Additional file 6: Figure S3. Diagnostic accuracy of index test EUA in the diagnosis of partial and complete ACL injury. Legend: Risk of bias judgements: (−) = high risk; (?) = unclear risk; (+) = low risk. LR thresholds: +LR <5 and –LR >0.2 = small; +LR 5–10 and; −LR 0.1–0.2 = moderate and +LR>10 and –LR <1 = large. Studies that reported estimates for complete ACL injury as well as partial and complete ACL injury estimates have been plotted together to provide a comparison of test performance. Different symbols are used for the estimates for complete versus partial and complete ACL injury and for primary care versus secondary contact settings. Guide for interpretation: Greater distance between the –LR and +LR symbols for the test indicates better diagnostic performance. Click here for file
