# Supplementary material for: Accuracy of clinical tests in the diagnosis of anterior cruciate ligament injury: a systematic review
Source: Chiropr Man Therap. 2014 Aug 1;22:25. doi: 10.1186/s12998-014-0025-8 (PMC4152763; doi:10.1186/s12998-014-0025-8)
Supplement: Additional file 1: Table S1. — PubMed search strategy. [file s12998-014-0025-8-S1.pdf]

Table S1. PubMed search strategy

**1. Index test:**
"Medical History Taking"[mesh] OR history[tw] OR Pain[mesh] OR pain[tw] OR complaint*[tw] OR dysfunction*[tw] OR disabil*[tw] OR "Physical Examination" [mesh] OR "physical examination"[tw] OR "function test"[tw] OR "physical test"[tw] OR ((clinical[tw] OR clinically[tw]) AND (diagnosis[tw] OR sign[tw] OR signs[tw] OR significance[tw] OR symptom*[tw] OR parameter*[tw] OR assessment[tw] OR finding*[tw] OR evaluat*[tw] OR indication*[tw] OR examination*[tw])) OR "Joint instability"[mesh] OR "give way"[tw] OR pop[tw] OR "lachman test"[tw] OR "anterior drawer test"[tw] OR "pivot shift test"[tw]
**2. Target condition:**
"Anterior Cruciate Ligament"[mesh] OR "ACL"[tw] OR "anterior cruciate"[tw] OR "Cruciate"[tw]
**3. Exclusion criteria:**
(Animals[mesh] NOT (Animals[mesh] AND Humans[mesh])) NOT "case report"[ti]
**4. Search combination**
1 AND 2 NOT 3
