# Supplementary material for: Accuracy of clinical tests in the diagnosis of anterior cruciate ligament injury: a systematic review
Source: Chiropr Man Therap. 2014 Aug 1;22:25. doi: 10.1186/s12998-014-0025-8 (PMC4152763; doi:10.1186/s12998-014-0025-8)
Supplement: Additional file 4: Figure S1. — Anterior draw, Lachman and pivot shift tests plotted on the ROC plane. Legend: Green: Primary care study setting. Red: Secondary contact study setting. [file s12998-014-0025-8-S4.pdf]

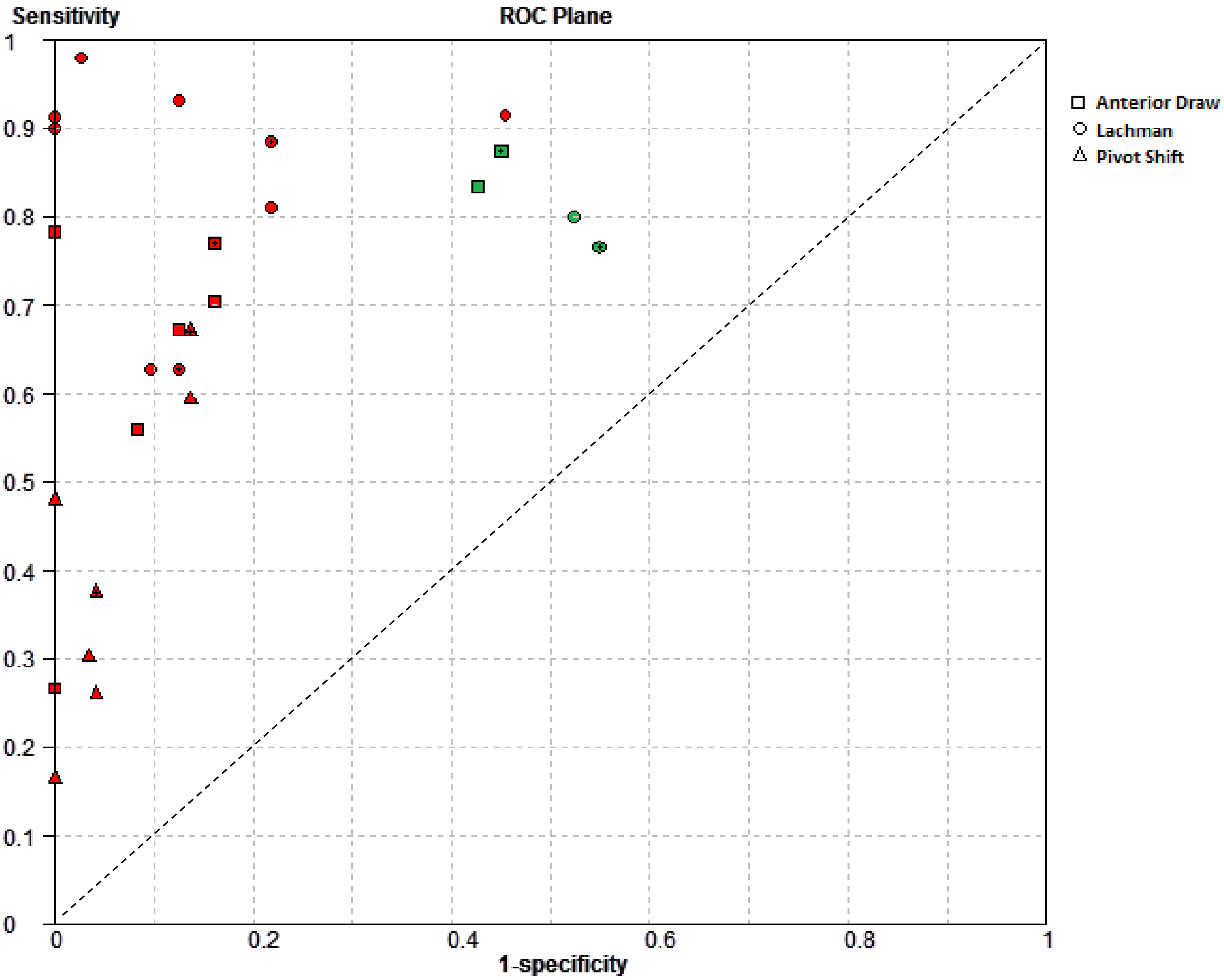

Sensitivity
ROC Plane
1
0.9
0.8
0.7
0.6
0.5
0.4
0.3
0.2
0.1
0
0
0.2
0.4
0.6
0.8
1
1-specificity
Anterior Draw
Lachman
Pivot Shift

Anterior Draw

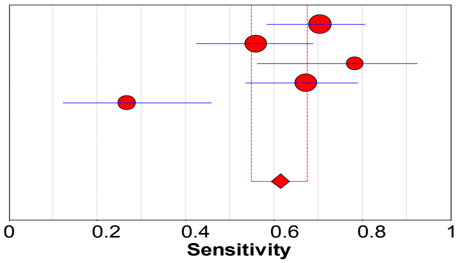

	Sensitivity (95% CI)	
Beldame. 2011	0.70	(0.58 - 0.81)
Boeree. 1991	0.56	(0.42 - 0.69)
Lee. 1988	0.78	(0.56 - 0.93)
Richter. 1996	0.67	(0.54 - 0.79)
Tonino. 1986	0.27	(0.12 - 0.46)

Pooled Sensitivity = 0.61 (0.55 to 0.68)
Chi-square = 22.00; df = 4 (p = 0.0002)
Inconsistency (I-square) = 81.8 %

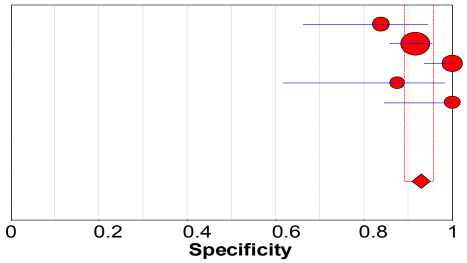

	Specificity (95% CI)	
Beldame. 2011	0.84	(0.66 - 0.95)
Boeree. 1991	0.92	(0.86 - 0.96)
Lee. 1988	1.00	(0.94 - 1.00)
Richter. 1996	0.88	(0.62 - 0.98)
Tonino. 1986	1.00	(0.85 - 1.00)

Pooled Specificity = 0.93 (0.89 to 0.96)
Chi-square = 15.28; df = 4 (p = 0.0042)
Inconsistency (I-square) = 73.8 %

Lachman

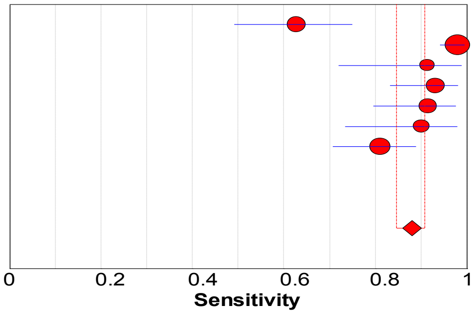

	Sensitivity (95% CI)	
Boeree. 1991	0.63	(0.49 - 0.75)
Harilainen. 1987	0.98	(0.94 - 1.00)
Lee. 1988	0.91	(0.72 - 0.99)
Richter. 1996	0.93	(0.83 - 0.98)
Schwarz. 1997	0.91	(0.80 - 0.98)
Tonino. 1986	0.90	(0.73 - 0.98)
Beldame. 2011	0.81	(0.71 - 0.89)

Pooled Sensitivity = 0.88 (0.85 to 0.91)
Chi-square = 50.66; df = 6 (p = 0.0000)
Inconsistency (I-square) = 88.2 %

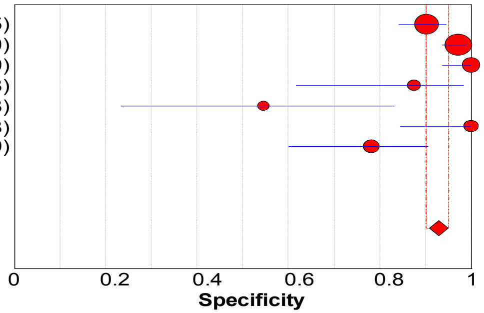

	Specificity (95% CI)	
Boeree. 1991	0.90	(0.84 - 0.95
Harilainen. 1987	0.97	(0.94 - 0.99
Lee. 1988	1.00	(0.94 - 1.00
Richter. 1996	0.88	(0.62 - 0.98
Schwarz. 1997	0.55	(0.23 - 0.83
Tonino. 1986	1.00	(0.85 - 1.00
Beldame. 2011	0.78	(0.60 - 0.91

Pooled Specificity = 0.93 (0.90 to 0.95)
Chi-square = 39.41; df = 6 (p = 0.0000)
Inconsistency (I-square) = 84.8 %

Pivot Shift

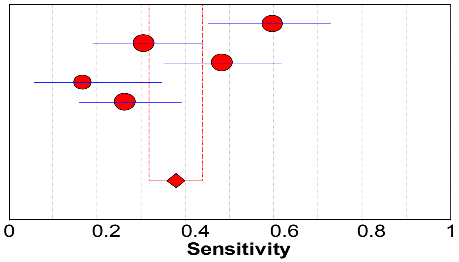

	Sensitivity (95% CI)	
Beldame. 2011	0.60	(0.45 - 0.73)
Boeree. 1991	0.31	(0.19 - 0.44)
Richter. 1996	0.48	(0.35 - 0.62)
Tonino. 1986	0.17	(0.06 - 0.35)
Noyes. 1980	0.26	(0.16 - 0.39)

Pooled Sensitivity = 0.38 (0.32 to 0.44)
Chi-square = 24.21; df = 4 (p = 0.0001)
Inconsistency (I-square) = 83.5 %

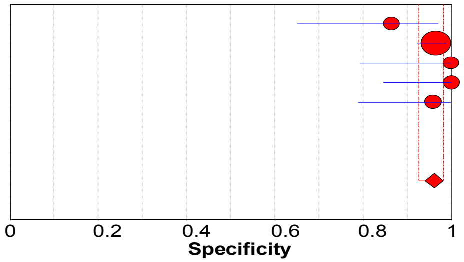

	Specificity (95% CI)	
Beldame. 2011	0.86	(0.65 - 0.97)
Boeree. 1991	0.97	(0.92 - 0.99)
Richter. 1996	1.00	(0.79 - 1.00)
Tonino. 1986	1.00	(0.85 - 1.00)
Noyes. 1980	0.96	(0.79 - 1.00)

Pooled Specificity = 0.96 (0.93 to 0.98)
Chi-square = 6.55; df = 4 (p = 0.1616)
Inconsistency (I-square) = 38.9 %

Anterior Draw

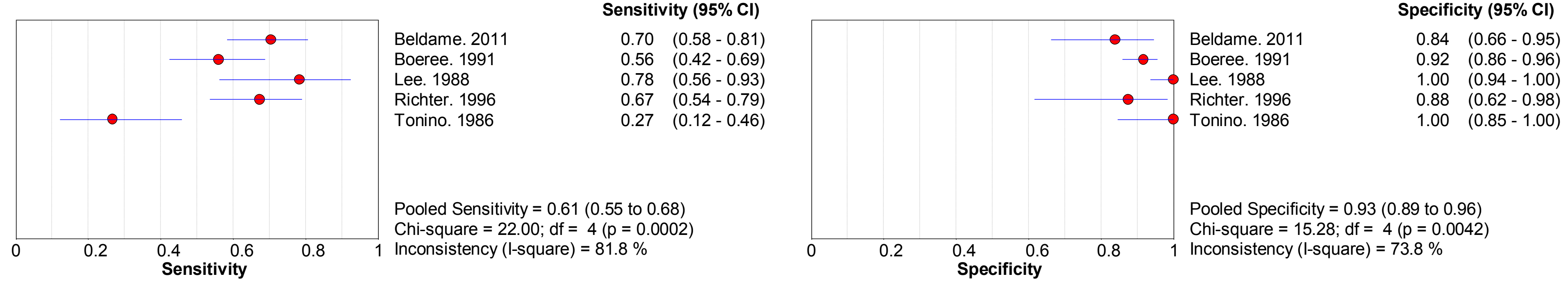

Lachman

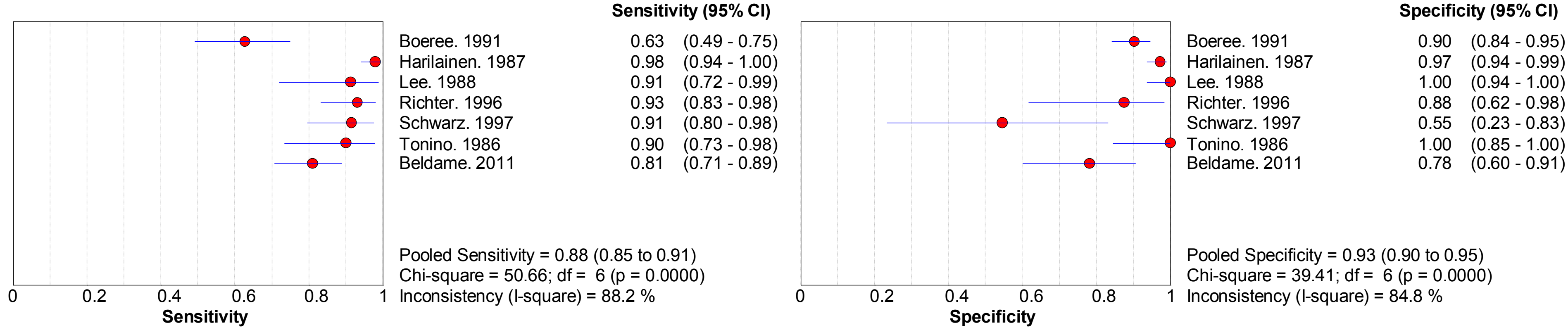

Pivot Shift

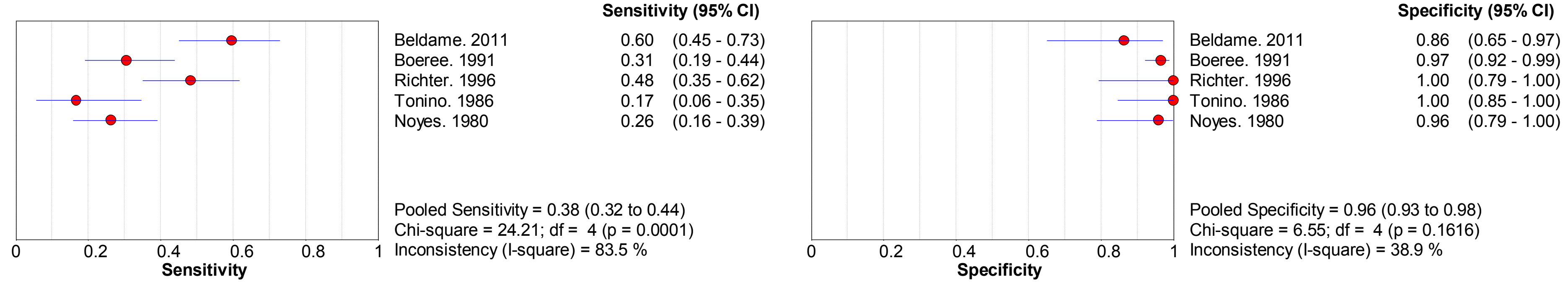

Anterior Draw

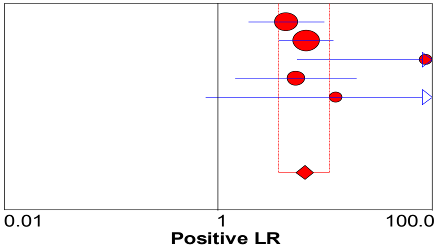

	Positive LR (95% CI)	
Beldame. 2011	4.37	(1.93 - 9.88)
Boeree. 1991	6.71	(3.73 - 12.07)
Lee. 1988	87.87	(5.52 - 1,399.90)
Richter. 1996	5.38	(1.45 - 19.91)
Tonino. 1986	12.61	(0.77 - 207.56)

Random Effects Model
Pooled Positive LR = 6.40 (3.71 to 11.03)
Cochran-Q = 4.90; df = 4 (p = 0.2975)
Inconsistency (I-square) = 18.4 %
Tau-squared = 0.0749

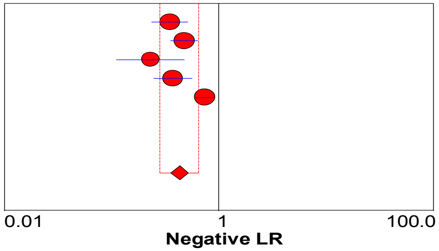

	Negative LR (95% CI	
Beldame. 2011	0.35	(0.24 - 0.52)
Boeree. 1991	0.48	(0.36 - 0.64)
Lee. 1988	0.23	(0.11 - 0.48)
Richter. 1996	0.37	(0.25 - 0.57)
Tonino. 1986	0.74	(0.59 - 0.93)

Random Effects Model
Pooled Negative LR = 0.43 (0.28 to 0.65)
Cochran-Q = 26.34; df = 4 (p = 0.0000)
Inconsistency (I-square) = 84.8 %
Tau-squared = 0.1789

Lachman

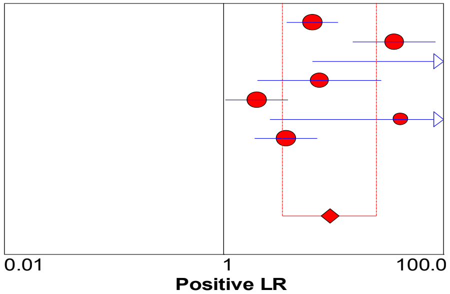

	Positive LR (95% CI)	
Boeree. 1991	6.45	(3.78 - 11.02)
Harilainen. 1987	35.65	(15.02 - 84.65)
Lee. 1988	102.13	(6.44 - 1,618.41)
Richter. 1996	7.45	(2.03 - 27.28)
Schwarz. 1997	2.01	(1.05 - 3.87)
Tonino. 1986	40.81	(2.62 - 634.75)
Beldame. 2011	3.70	(1.91 - 7.19)

Random Effects Model
Pooled Positive LR = 9.21 (3.44 to 24.70)
Cochran-Q = 45.92; df = 6 (p = 0.0000)
Inconsistency (I-square) = 86.9 %
Tau-squared = 1.3200

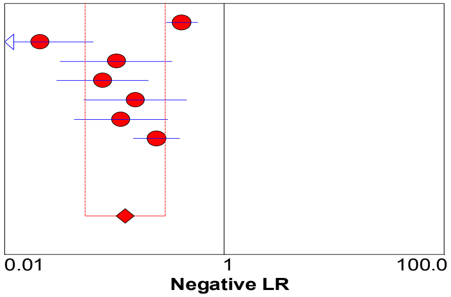

	Negative LR (95% CI	
Boeree. 1991	0.41	(0.30 - 0.58)
Harilainen. 1987	0.02	(0.01 - 0.06)
Lee. 1988	0.11	(0.03 - 0.34)
Richter. 1996	0.08	(0.03 - 0.21)
Schwarz. 1997	0.16	(0.05 - 0.46)
Tonino. 1986	0.12	(0.04 - 0.31)
Beldame. 2011	0.24	(0.15 - 0.40)

Random Effects Model
Pooled Negative LR = 0.13 (0.05 to 0.29)
Cochran-Q = 54.14; df = 6 (p = 0.0000)
Inconsistency (I-square) = 88.9 %
Tau-squared = 1.0629

Pivot Shift

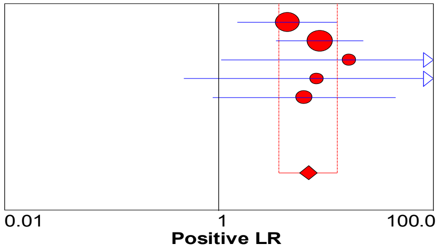

	Positive LR (95% CI)	
Beldame. 2011	4.37	(1.49 - 12.81)
Boeree. 1991	8.79	(3.42 - 22.57)
Richter. 1996	16.42	(1.06 - 255.24)
Tonino. 1986	8.16	(0.47 - 140.30)
Noyes. 1980	6.30	(0.88 - 44.88)

Random Effects Model
Pooled Positive LR = 6.87 (3.65 to 12.92)
Cochran-Q = 1.36; df = 4 (p = 0.8510)
Inconsistency (I-square) = 0.0 %
Tau-squared = 0.0000

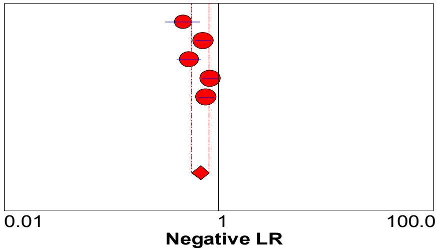

	Negative LR (95% CI	
Beldame. 2011	0.47	(0.32 - 0.68)
Boeree. 1991	0.72	(0.61 - 0.85)
Richter. 1996	0.53	(0.41 - 0.69)
Tonino. 1986	0.84	(0.71 - 1.00)
Noyes. 1980	0.77	(0.65 - 0.91)

Random Effects Model
Pooled Negative LR = 0.68 (0.56 to 0.81)
Cochran-Q = 15.12; df = 4 (p = 0.0045)
Inconsistency (I-square) = 73.5 %
Tau-squared = 0.0311

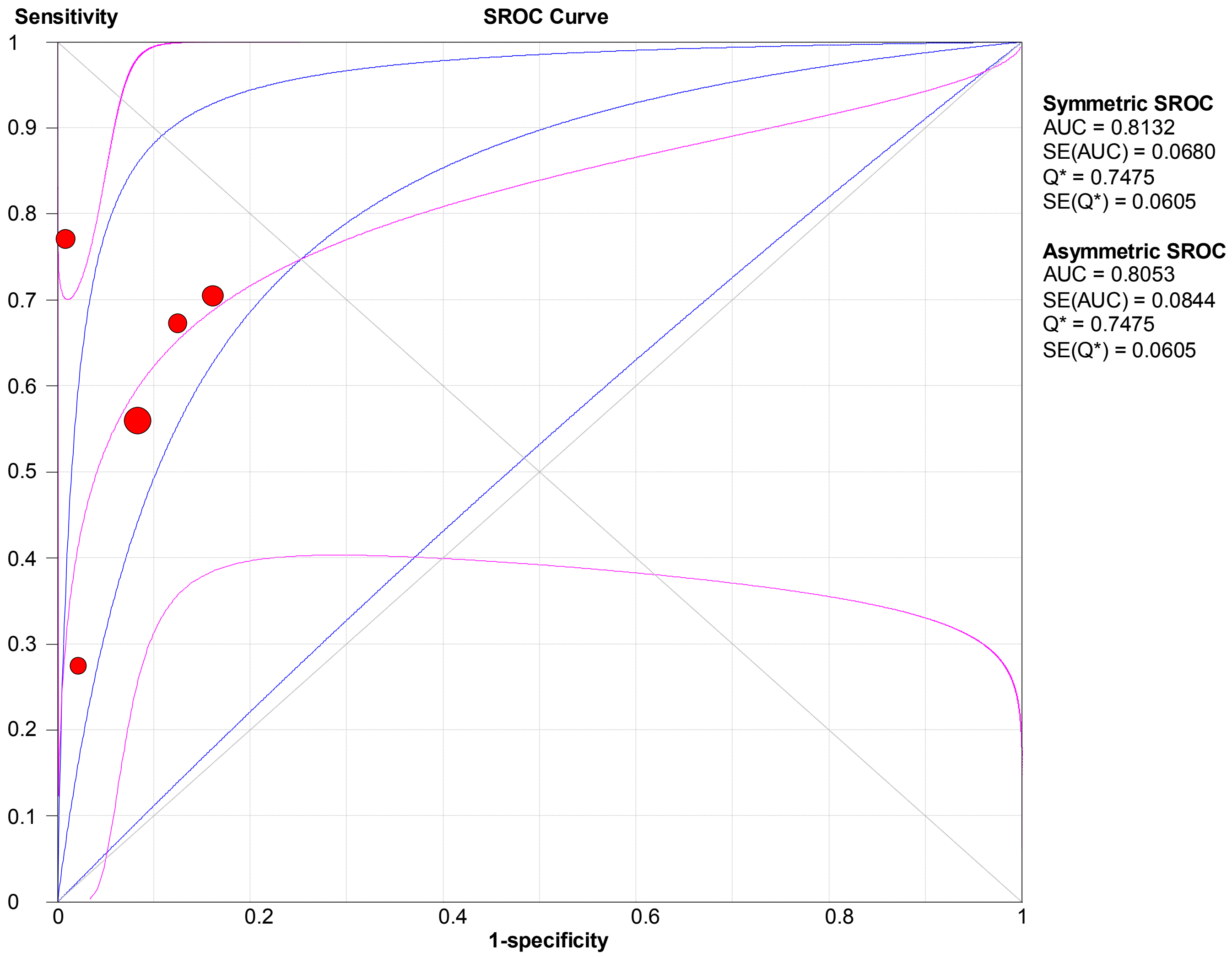
Sensitivity
SROC Curve
1
0.9
0.8
0.7
0.6
0.5
0.4
0.3
0.2
0.1
0
0
0.2
0.4
0.6
0.8
1
1-specificity
Symmetric SROC
AUC = 0.8132
SE(AUC) = 0.0680
Q* = 0.7475
SE(Q*) = 0.0605
Asymmetric SROC
AUC = 0.8053
SE(AUC) = 0.0844
Q* = 0.7475
SE(Q*) = 0.0605

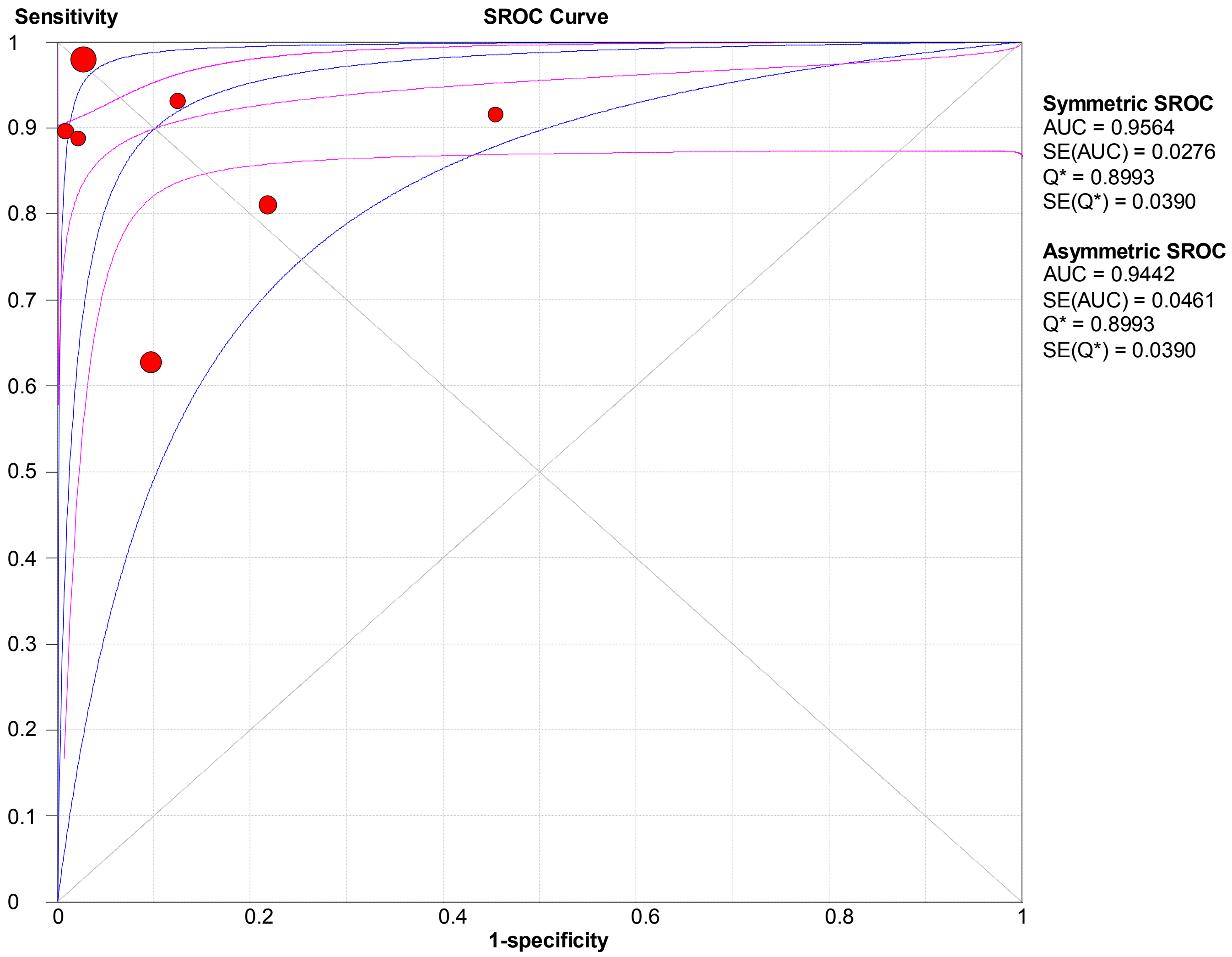
Sensitivity
SROC Curve
1
0.9
0.8
0.7
0.6
0.5
0.4
0.3
0.2
0.1
0
0
0.2
0.4
0.6
0.8
1
1-specificity
Symmetric SROC
AUC = 0.9564
SE(AUC) = 0.0276
Q* = 0.8993
SE(Q*) = 0.0390
Asymmetric SROC
AUC = 0.9442
SE(AUC) = 0.0461
Q* = 0.8993
SE(Q*) = 0.0390

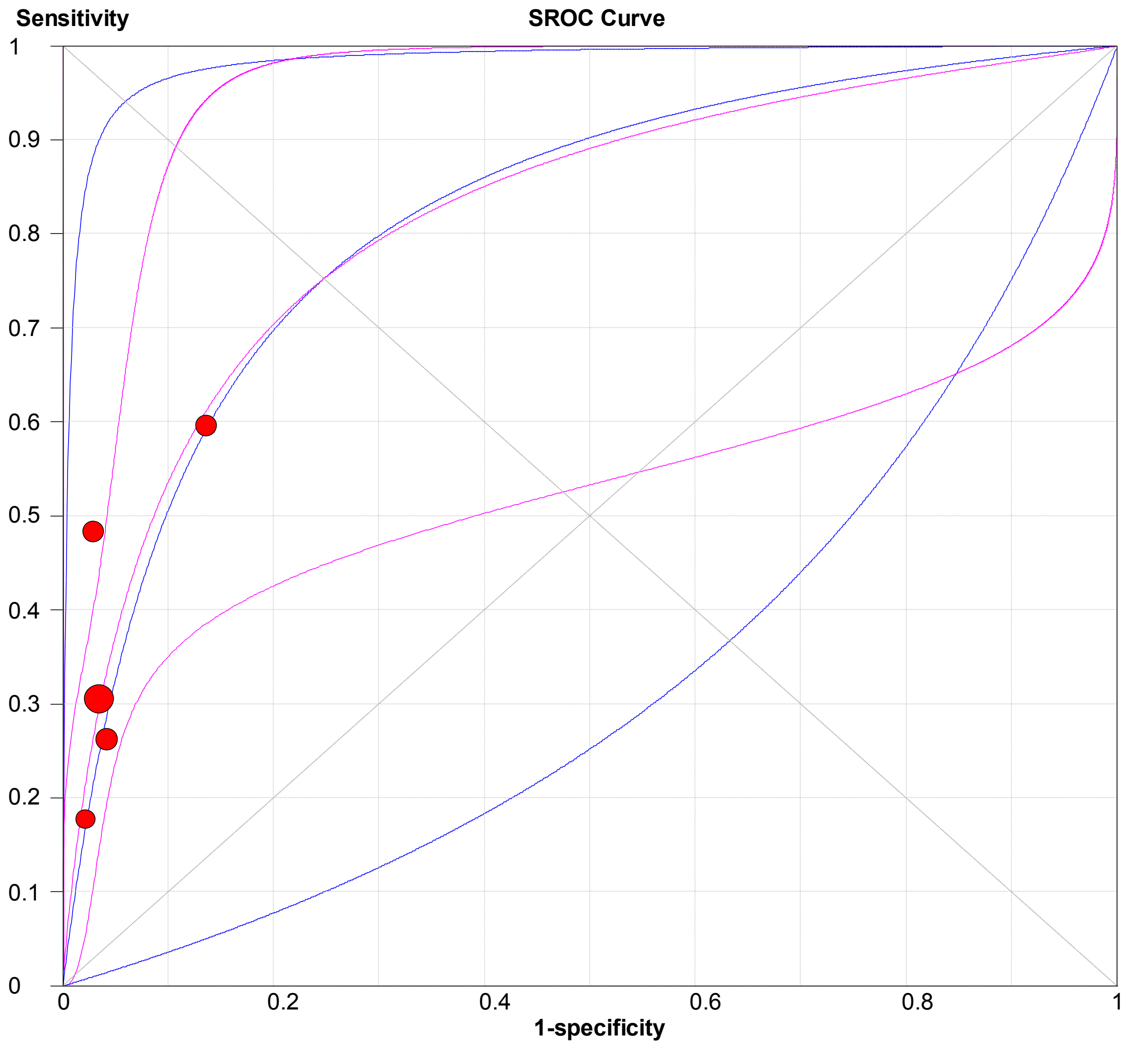

Symmetric SROC
AUC = 0.8186
SE(AUC) = 0.1081
Q* = 0.7524
SE(Q*) = 0.0969

Asymmetric SROC
AUC = 0.8184
SE(AUC) = 0.1086
Q* = 0.7524
SE(Q*) = 0.0969
