# Supplementary figures and images for: Accuracy of clinical tests in the diagnosis of anterior cruciate ligament injury: a systematic review
Source: Chiropr Man Therap. 2014 Aug 1;22:25. doi: 10.1186/s12998-014-0025-8 (PMC4152763; doi:10.1186/s12998-014-0025-8)

Anterior Draw

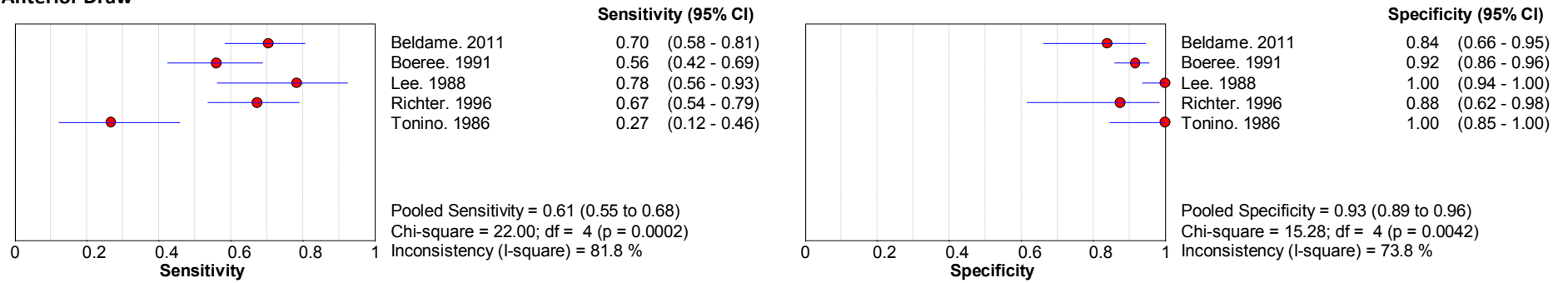

Lachman

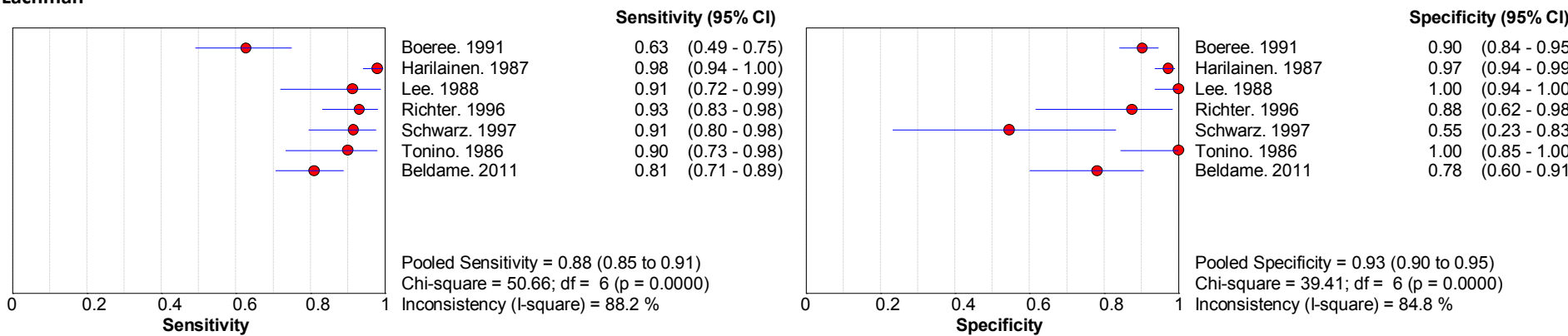

Pivot Shift

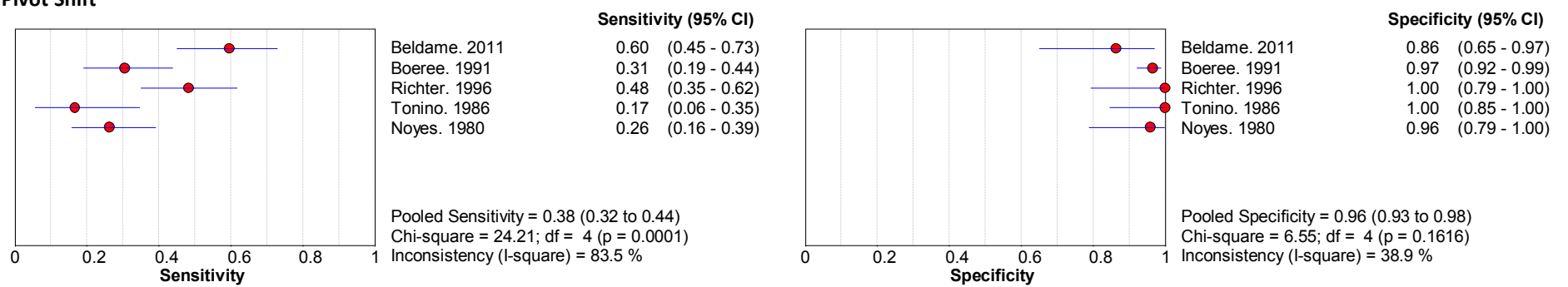

Supplement: Additional file 5: Figure S2. — Subgroup sensitivity and specificity forest plots. Legend: Anterior draw, Lachman and pivot shift test sensitivity and specificity for partial and complete ACL injury in secondary contact settings. [file s12998-014-0025-8-S5.pdf]

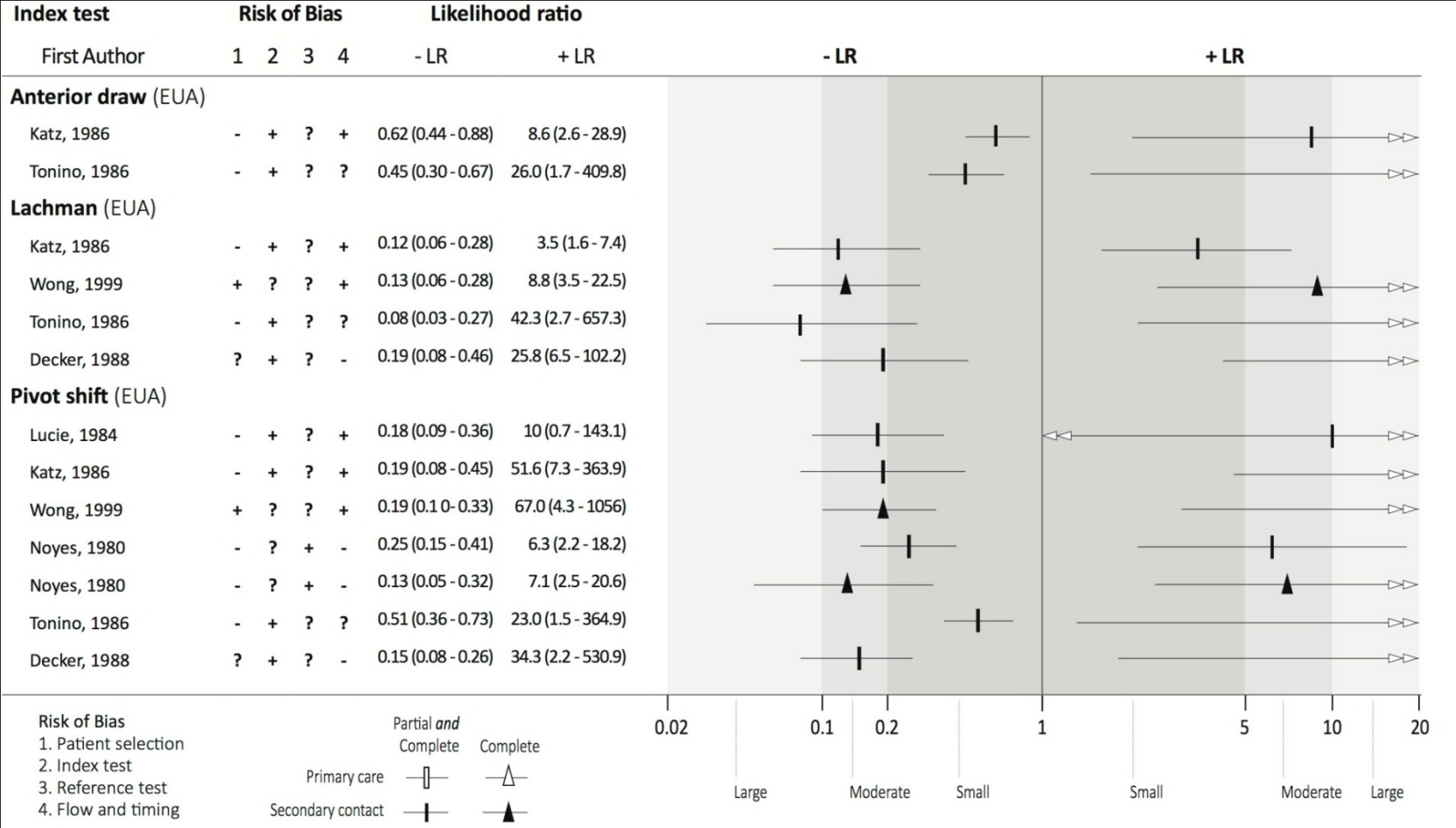

Supplement: Additional file 6: Figure S3. — Diagnostic accuracy of index test EUA in the diagnosis of partial and complete ACL injury. Legend: Risk of bias judgements: (−) = high risk; (?) = unclear risk; (+) = low risk. LR thresholds: +LR <5 and –LR >0.2 = small; +LR 5–10 and; −LR 0.1–0.2 = moderate and +LR>10 and –LR <1 = large. Studies that reported estimates for complete ACL injury as well as partial and complete ACL injury estimates have been plotted together to provide a comparison of test performance. Different symbols are used for the estimates for complete versus partial and complete ACL injury and for primary care versus secondary contact settings. Guide for interpretation: Greater distance between the –LR and +LR symbols for the test indicates better diagnostic performance. [file s12998-014-0025-8-S6.pdf]
